# An integrated model to study varietal diversity in traditional agroecosystems

**DOI:** 10.1371/journal.pone.0263064

**Published:** 2022-01-28

**Authors:** Vitor Hirata Sanches, Cristina Adams, Fernando Fagundes Ferreira

**Affiliations:** 1 School of Arts, Sciences and Humanities, University of São Paulo, São Paulo, SP, Brazil; 2 Institute of Energy and Environment, University of São Paulo, São Paulo, SP, Brazil; 3 Department of Physics-FFCLRP, University of São Paulo, Ribeirão Preto, SP, Brazil; International Centre for Integrated Mountain Development (ICIMOD), Kathmandu, Nepal, NEPAL

## Abstract

Agricultural diversity is one of the bases of traditional agroecosystems, having great environmental and cultural importance. The current loss of agricultural diversity is causing serious concern, mainly because of its essential role in supporting global and local food security. Stopping this loss requires a better understanding of how diversity is managed locally and what mechanisms sustain agricultural diversity. Here we propose a generalist agent-based model that couples biological, cultural, and social dynamics to obtain varietal diversity as an emergent phenomenon at the community level. With a mechanistic approach, we explore how four of the model dynamics can shape systems diversity. To validate the model, we performed a bibliographic review on *Manihot esculenta* and *Zea mays* case studies. The model yielded compatible results for manioc and maize varietal richness at both community and household levels.

## Introduction

The livelihoods of traditional and indigenous communities around the world are facing rapid changes, including the loss of agricultural diversity [1–3]. The academic and development literature have given increasing attention to this fact due to its impact on food security and genetic diversity, which are crucial in a world facing climate change [1–4]. Thus, there is a need to better understand the origins of agrobiodiversity and the social and biological mechanisms that create and sustain it.

Agrobiodiversity is a broad term that incorporates all components of biodiversity relevant to agroecosystems, helping to sustain their key functions, structure, and processes [5]. These components include the variety and variability of animals, plants, and microorganisms at the genetic, species, and ecosystem levels [5]. This type of diversity results from interactions between genetic resources, the environment, and the culturally diverse practices and management systems adopted by people in socioecological systems [6]. The present study focuses on a subcomponent of agrobiodiversity, the varietal diversity. A variety is defined as the smaller unit of perception and management of agricultural diversity [7].

Numerous traditional agroecosystems around the world have high levels of varietal diversity [8], especially the ones situated in the so-called centers of crop diversity (e.g., Southern Mexico for maize [9] and southwestern Amazonia for manioc [10]). Varietal diversity increases the resilience of agricultural systems by buffering crop production from climate variability and acting as a pest and disease control mechanism [2, 11, 12]. For example, varietal diversity can create biotic barriers against diseases, as mixtures of susceptible and resistant varieties can decrease disease propagation [11]. Maintaining high varietal diversity is also an important risk-reducing management strategy that mitigates annual and seasonal variations in crop production [12–15]. This strategy is also referred to in the literature as the portfolio effect, a concept brought from financial assets management that has been applied to ecology [16].

Due to intensification of land use, market influence, and public incentives for farm modernization, agricultural diversity is under severe threat [1]. Studies demonstrate that varietal diversity is decreasing in many places [1, 3, 17]. According to an FAO report of 2010, up to 60 countries report genetic vulnerability in agricultural production, with the main cause of genetic erosion being the substitution of local varieties for genetically uniform, high-yielding varieties [1]. Although a global analysis shows that farmers maintain a high number of varieties [18], the current global trend of agricultural varietal diversity is still poorly documented [19].

Besides the major threats to global and local food security, the loss of agrobiodiversity can also have socioecological impacts by reducing the ecosystem services it provides (e.g., pollination, pest and disease control, evolutionary services) [9, 20, 21]. Agrobiodiversity is a human cultural heritage [22] and a key public good [12]. Initiatives such as the FAO Globally Important Agricultural Heritage Systems (GIAHS) consider traditional agricultural systems as living, evolving systems of human communities that interact with their territorial, cultural, and agricultural landscapes. This shows the importance of these socioecological systems for in situ conservation of agrobiodiversity (http://www.fao.org/giahs/background/en/).

Many indigenous and traditional communities have independently developed highly diverse agricultural systems from individual farmer’s management strategies that increase varietal diversity [8, 14]. The literature explains the existence of high varietal diversity in different ways, using biologic, socioeconomic, and cultural parameters. The high diversity could be a consequence of biological factors such as environmental variability and heterogeneity [23, 24]; socioeconomic factors such as a strategy to cope with risk, bringing resilience to agricultural systems and assuring food security [11–14] or labor availability and organization [25]; or, finally, cultural factors such as the multiple uses of agricultural varieties [26, 27], cultural and linguistic diversity [28, 29], culinary preferences [25], or simply the pleasure of collecting and experiencing new varieties [23, 30, 31]. However, we still do not have a general socioecological framework to explain the different ways by which individual management of varietal diversity leads to systems diversity at the community/village level.

So, the question we address here is how can local interactions among heterogeneous autonomous agents—farmers—generate high varietal diversity at the community level? Answering this generative question [32] can improve understanding of the origins of varietal diversity and the social and biological dynamics that create and sustain it. To address this question, we developed a multilevel agent-based model that integrates plot (or farm), household, and community levels to study varietal diversity.

Models have been an important integrative tool in social-ecological systems research. They can facilitate the integration of disciplinary knowledge and help understanding how human-environmental systems are coupled [33]. Different model approaches can serve different purposes, ranging from simple and generic dynamical system models—used to capture general system behavior—to complicated and realistic agent-based models—used to predict outcomes of different scenarios [33]. We used a simple stylized agent-based model as a way to test assumptions and gain insights about the system’s behavior, clarifying the core mechanisms responsible for sustaining varietal diversity. Formulating generalist agent-based models is also a way to illuminate the core dynamics of a system, understand emergent behavior, and help theory building in early stages of a particular problem [33, 34], contributing to the development of more realistic models in the future.

Despite these benefits, to date, only a few models have been developed to study varietal diversity. [35, 36] use dynamical systems models derived from epidemiologic models to simulate the introduction of varieties in social networks. [36] analyze the effect of different network topologies on the diffusion and persistence of introduced varieties. These models were applied to case studies and uncover important knowledge on the role of the seed exchange network. However, they consider a simplified situation consisting of the diffusion of one variety within a network of homogeneous households. Additionally, these dynamical systems models did not consider household agency and heterogeneity [33], which makes these models incapable of answering the broader question posed here.

[37] built a participatory agent-based model of sorghum varietal diversity in Mali. The authors obtained predictions at the community level for different scenarios of public interventions and climate conditions. They successfully address multiple levels within a community and integrate disciplinary knowledge from experts and local households. However, their model does not account for the creation of new varieties (the study used only previously created and parameterized varieties) and requires a large amount of empirical data. Our model differs as it focuses on answering a more theoretical question of which core mechanisms promote agricultural varietal diversity, with a simpler and more generalist approach.

To validate our model, we used case studies of *Manihot esculenta* Crantz. (manioc or cassava) and *Zea mays* ssp. *mays* (maize) varietal diversity in traditional agricultural systems. Both species are among the most important staple foods in their centers of domestication, having an enormous varietal diversity and being central to archaeological theories of human occupation of the Americas [38, 39]. Manioc is a tuberous root rich in starch and an important cultivar for indigenous communities in the Amazon, being one of their major energy sources [22, 40]. It was probably domesticated in the savannahs near southern Amazon between 8,000–10,000 BP [38, 41]. An FAO report considered the existence of 7,000 varieties of manioc worldwide [42], with 29–129 varieties occurring in different indigenous communities in the Amazon [25, 31, 43]. Manioc varieties are distinguished in the Amazon from the size and architecture of the overground part of the plant, followed by the color of the petiole and the tuber characteristics. The names are an essential attribute of the varieties and usually refer to overground part characteristics [44, 45]. In the Amazon, indigenous people value manioc varietal diversity, which is created by social norms, social networks for germplasm exchange, and the management of sexual propagation [31, 44, 46, 47]. Although vegetative propagation is the main cultivation strategy, manioc can reproduce sexually, which plays a very important role in the evolutionary dynamics of the crop [31, 41, 48].

Maize is a cereal grain rich in starch and is the crop with the widest global distribution due to its environmental adaptability and productivity. Maize destined for direct human consumption is mostly produced by smallholders that manage high genetic diversity under a wide range of different socioecological conditions. Mexico is the center of origin for maize (circa 9,000 BP) and one of its centers of diversity. The country hosts 59–60 different varieties, mostly cultivated by smallholders [9, 49]. [50] report 26 named varieties of maize in Cuzalapa, an indigenous community in Western Mexico. In traditional systems, evolution continues to actively shape genetic diversity due to cultural practices such as smallholders saving their own seeds, growing several varieties in the same plot, and tolerating sympatric growth of teosinte subspecies. These practices create diverse opportunities for gene flow through open pollination with neighboring plants [9]. Historical diffusion of maize out of the Americas led to the selection of hundreds of new varieties in the past 500 years [51].

## Materials and methods

### The proposed model

**Fig 1:**
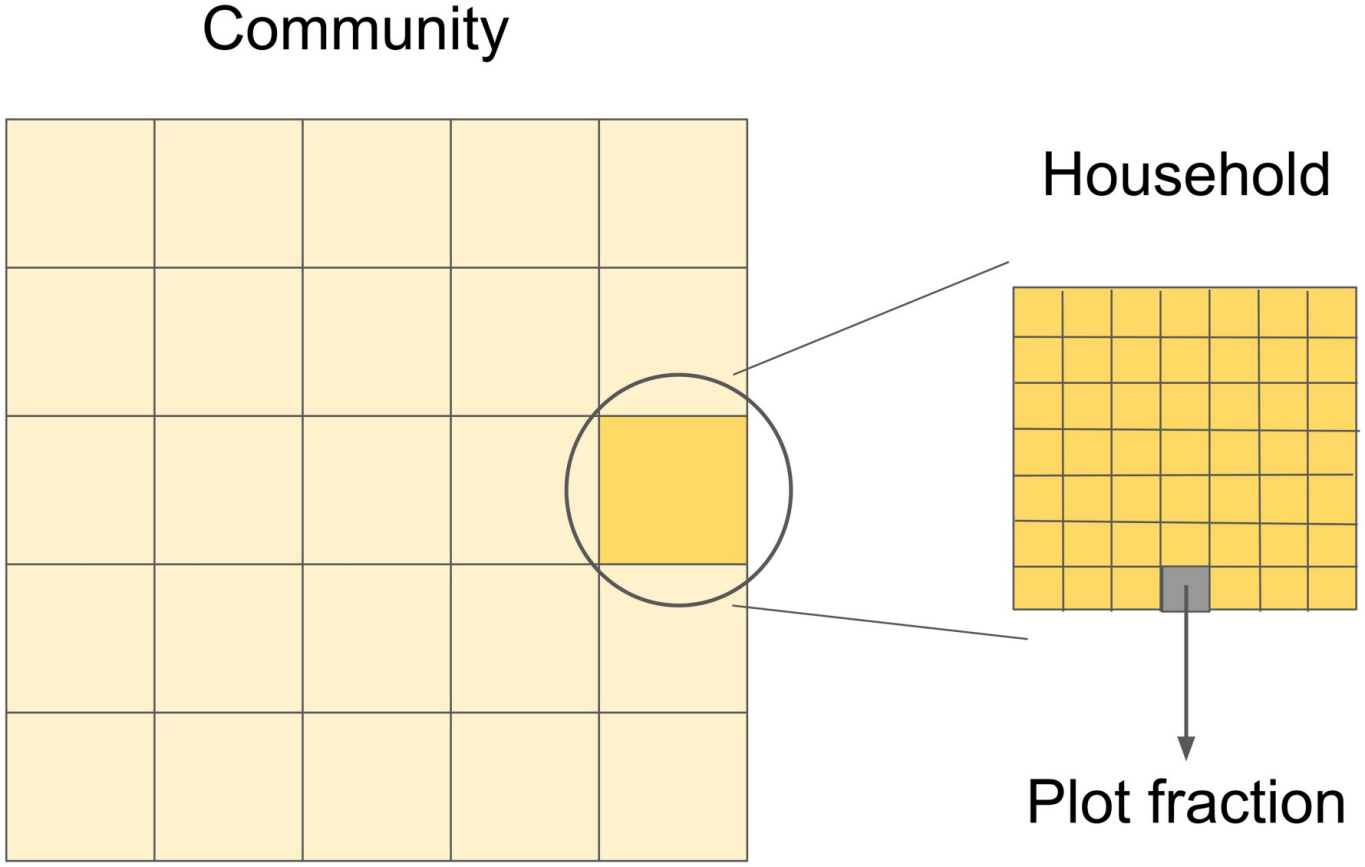
Diagram with the three model levels: Community, household, and plot fraction.

A multilevel model was developed, grouping plot fraction, household, and community/village levels (Fig 1) [12, 52]. The main unit of analysis is the community macro level. It represents a small, relatively isolated traditional community, with a few dozen households (HDs). The household is the micro level unit where the dynamics of each agent is defined. It represents a coresident domestic group that is responsible for the agricultural production of the unit [53]. The smaller level of the model is the plot fraction. A household owns one plot, divided in multiple plot fractions, where individual varieties are cultivated.

In addition to these three elements—community, household, and plot fraction –, we added a fourth one—variety. A variety is determined here by a set of half-saturation constants—a parameter used to measure productivity—and quality—a parameter used to measure household’s quality fit. Table 1 shows a summary of the four elements of the model.

**Table 1:** Description of the model’s four elements: Land fragment, household, community and variety.

Element	Description
Community	Group of households.
Household	Controls which crop varieties will be planted in the plot. A household is linked to other households, with whom it can exchange varieties.
Plot fraction	Contains some fixed amount of biochemical resources (nitrogen, phosphorus, potassium, and water) and one crop variety.
Variety	Variety of a food crop, defined by its half-saturation constants and quality.

In designing the model, assumptions were made at the level of the household: (1) each household selects which variety and how much of it will be planted based on a variety suitability ranking that considers its productivity and quality fit; (2) each household chooses to cultivate a greater number of varieties that have a high score, keeping low-scoring varieties at low density; (3) each household exchanges varieties with other households of the community based on a fixed network, and prefers to exchange with high-scoring households. These concepts are explained below.

Household variety selection follows the criteria proposed by [8]. According to the author, selection criteria can be grouped into three categories: (i) *productivity*—an obvious and important criterion verified in the majority of case studies; (ii) *quality fit*—a broad concept that embraces taste, processing and cooking qualities, resistance to pests, spoilage during storage, and market demand; and (iii) *perceived risk* of crop failure. The *perceived risk* is harder to be quantified and has an intersection with productivity, thus not being used in the present study with a view to simplify the model.

*Productivity* or yield (mass per unit area and unit time) is determined by the adequacy of the variety to the environment. The Monod Equation [54, 55] was used to quantify productivity; thus, the productivity of an individual plant will depend only on the limiting resource available in its plot. Naturally, productivity/yield depends on multiple biochemical resources, on resource-independent variety traits (such as growth rate and proportion of energy allocated to different structures), and on neglected effects such as biotic and thermal stress. Nevertheless, the Monod Equation was used as a first order approximation due to its simplicity.

Quality was quantified as a real number in the interval [0, 1]. The *quality fit* is then calculated based on the distance between the desired quality of the HD (*q*_*HD*_) and the quality of the variety (*q*_*v*_). To avoid preferred qualities and maintain symmetry on quality, the distance is circular and therefore the maximum distance is 0.5. These two criteria embrace biophysical and cultural aspects of the selection criteria. They are coupled with a parameter *α*, a fixed global parameter of the model that weighs the importance of each criterion (Eq 1) Both criteria can be interpreted as dimensionless probabilities that remain within the interval [0, 1], with the same happening to the complete variety score (*s*_*v*,*HD*_).

$$\begin{array}{c} s_{v,HD}=\alpha \underset{\text{Productivity}}{\underset{︸}{\frac{R_{v}^{L}}{R_{v}^{L}+K_{v}^{L}}}}+\left(1-\alpha \right)\underset{\text{Quality}\;\text{fit}}{\underset{︸}{\left(1-2D\left(q_{v},q_{HD}\right)\right)}}. \end{array}$$ (1)

Where *s*_*v*,*HD*_ is the score of variety *v* in the household *HD*; *R*_*v*,*L*_ is the amount of the limiting resource for variety *v*—usually given as a function of the resources of each plot fraction, however, in this equation, we simplify using uniform resource distribution among plot fractions; *K*_*v*,*L*_ is the half-saturation constant of variety *v* for the limiting resource; and *D*(*x*, *y*) = *min*{|*x* − *y*|, |1 − (*x* − *y*)|} is the distance between two real values.

To decide which varieties will be planted in one cycle, the household considers the density and score of each variety. According to [23, p. 254] in a case study of the diversity of manioc amongst the Makushi, in Guiana, “a variety is rarely discarded, even if it is not very productive. Low-yielding, rare varieties are simply kept at low density (i.e. one or two mounds per farm)”. Therefore, in the model, the household (HD) seeks to maintain low-scoring varieties within few plot fractions, and high-scoring varieties within more plot fractions.

In each cycle, the HD keeps the majority of its production and exchanges up to 40% plot fractions varieties, with the exact number of exchanges determined randomly. Each household tries to minimize, in module, the difference between desired variety density ($\mu_{v,HD}^{d}$) and real variety density ($\mu_{v,HD}^{r}$). This difference $\Delta \mu_{v,HD}=\mu_{v,HD}^{d}-\mu_{v,HD}^{r}$ is interpreted as how much the HD wants to increase (or decrease) the production of a variety. In a cycle, the variety with higher Δ*μ*_*v*,*HD*_ will be cultivated in at least one more plot fraction, and the variety with lower Δ*μ*_*v*,*HD*_ will be cultivated in at least one less plot fraction. The real variety density is the fraction of the HD’s plot fractions where the variety is present. In turn, the calculation of the desired variety density is as follows in Eq 2:

$$\begin{array}{c} \mu_{v,HD}^{d}=\frac{f(s_{v,HD}-\bar{s_{HD}})}{\sum_{v}f\left(s_{v,HD}-\bar{s_{HD}}\right)}, \end{array}$$ (2)

where $\bar{s_{HD}}$ is the average score between the *HD*’s varieties, and *f* is a sigmoid renormalization function. The function *f* is used to increase the input range around $s_{v,HD}-\bar{s_{HD}}=0$, as this difference is small and usually stays within the interval [-0.2, 0.2]. For more details about this dynamics, see S1 Text.

Exchanges among households are considered to be donations and can only happen inside the community, between linked HDs. This fixed HD network is representative of kinship, godparenting, and neighborhood networks where the majority of exchanges occur [28, 31, 46, 56]. Though there is little evidence about the most appropriate HD network structure to describe traditional communities, a small-world network generated through the Watts-Strogatz model was used here because seed exchange networks seem to present small-world properties (local connectivity and some long-distance connections) [57]. All HDs have a common base probability of performing an exchange with each of the connected households. To compute the final exchange probability, the base value is weighted by the score of the aimed HD—average of owned varieties score, weighted by the density of each variety. Every time an exchange happens, an HD receives a variety from the selected HD. This variety is selected stochastically, with the probability of selection being the real variety density ($\mu_{v,HD}^{r}$).

**Fig 2:**
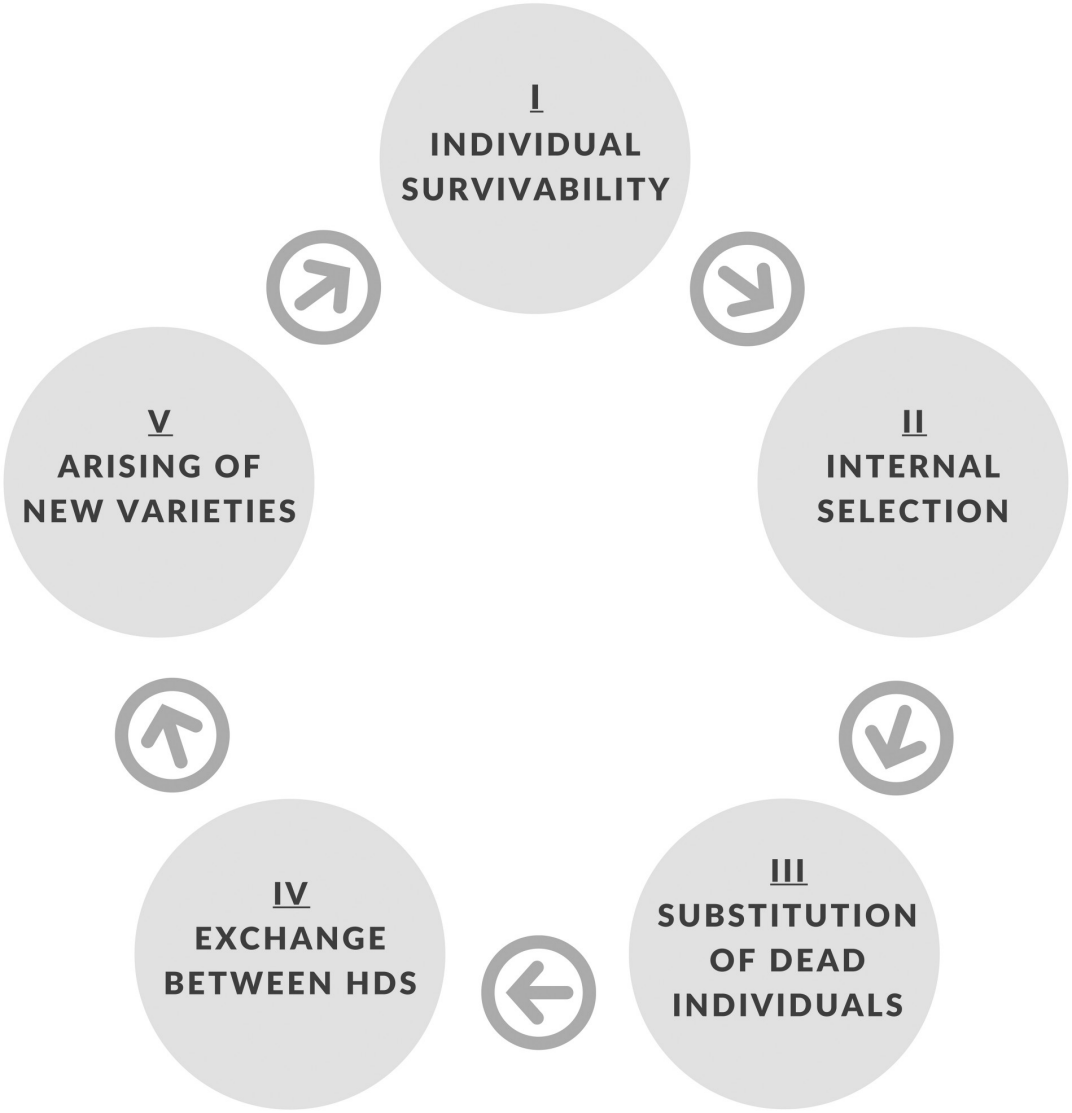
Flowchart of model steps.

One agricultural cycle of the model, that is, a complete process of planting-harvesting a food crop, has five steps (Fig 2). Besides the selection (Step II) and exchange of varieties (Step IV), three more processes occur. One individual specimen can die (Step I), which happens stochastically, with a higher probability for varieties with low productivity. The dead individuals are replaced by the variety that each HD wants to cultivate at higher densities, i.e., the variety that has greater Δ*μ*_*v*,*HD*_ (step III). Moreover, new varieties can arise (Step V), accounting for both sexual reproduction—when the new variety inherits parameters from its ancestors—and external introduction, for example from exchanges that happen outside of the community—when the new variety is initialized with random parameters.

The model was executed for 49 households in the community, and each HD controlled what was planted in a 7x7 grid of plot fractions. The resource was homogeneously distributed among the plot fractions (*H* = 1), with *α* = 0.6, so that productivity and quality fit score have a similar proportion in the total score. Model parameterization was intended to reproduce qualitative empirical diversity data, and no direct empirical parameters were used. For more details on the model dynamics and parameters, see S1 Text.

### Case studies

**Fig 3:**
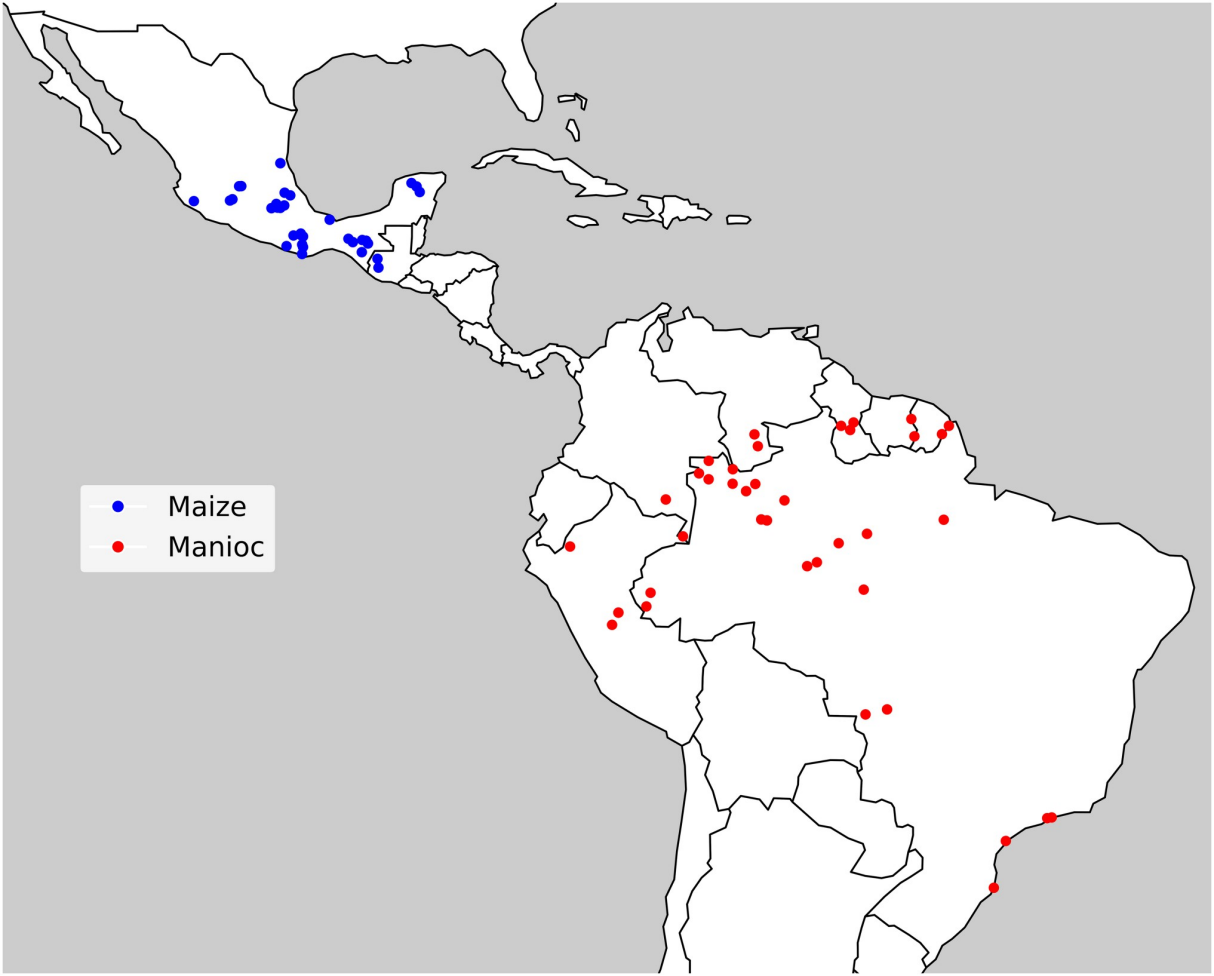
Locations of case studies included in the literature review. Each data point represents one case study location; articles can have more than one data point.

The authors conducted a literature review using the Web of Science database, applying the following keywords: “(cassava OR manioc) AND (landrace OR local variety OR folk) AND (diversity OR agrobiodiversity)” and “(maize) AND (landrace OR local variety OR folk) AND (diversity OR agrobiodiversity)”. The search was performed in May 2019, without restrictions on the year of publication. The Web of Science search yielded 80 articles for manioc and 521 for maize. Articles previously known by the authors and articles from earlier literature reviews were also added, namely, the reviews of [58] for manioc, and the ones of [59, 60] for maize. The results were filtered to consider only case studies with primary data on the number of named varieties per household or per community and within indigenous or traditional agroecosystems in South America (for manioc) and Central America (for maize). The authors selected a total of 22 papers for manioc and 33 for maize, with publication dates between 1959 and 2017. Fig 3 shows the location of the studies, and S1 Dataset shows the complete database.

The authors read the selected articles to help model development, also collecting quantitative data on varietal diversity. The collected data include: place and name of the communities, ethnicity, date of collection, number of informants, number of varieties in the community, average, standard deviation, minimum, and maximum number of varieties per household, evenness data—Simpson, Shannon, and Berger-Parker diversity indexes in both household and community levels –, and availability of detailed information on each household richness or the presence of the variety among households. Data on manioc and maize diversity were used to qualitatively fit the model; however, the parameterization derived to model manioc data was used in all images.

## Results

**Fig 4:**
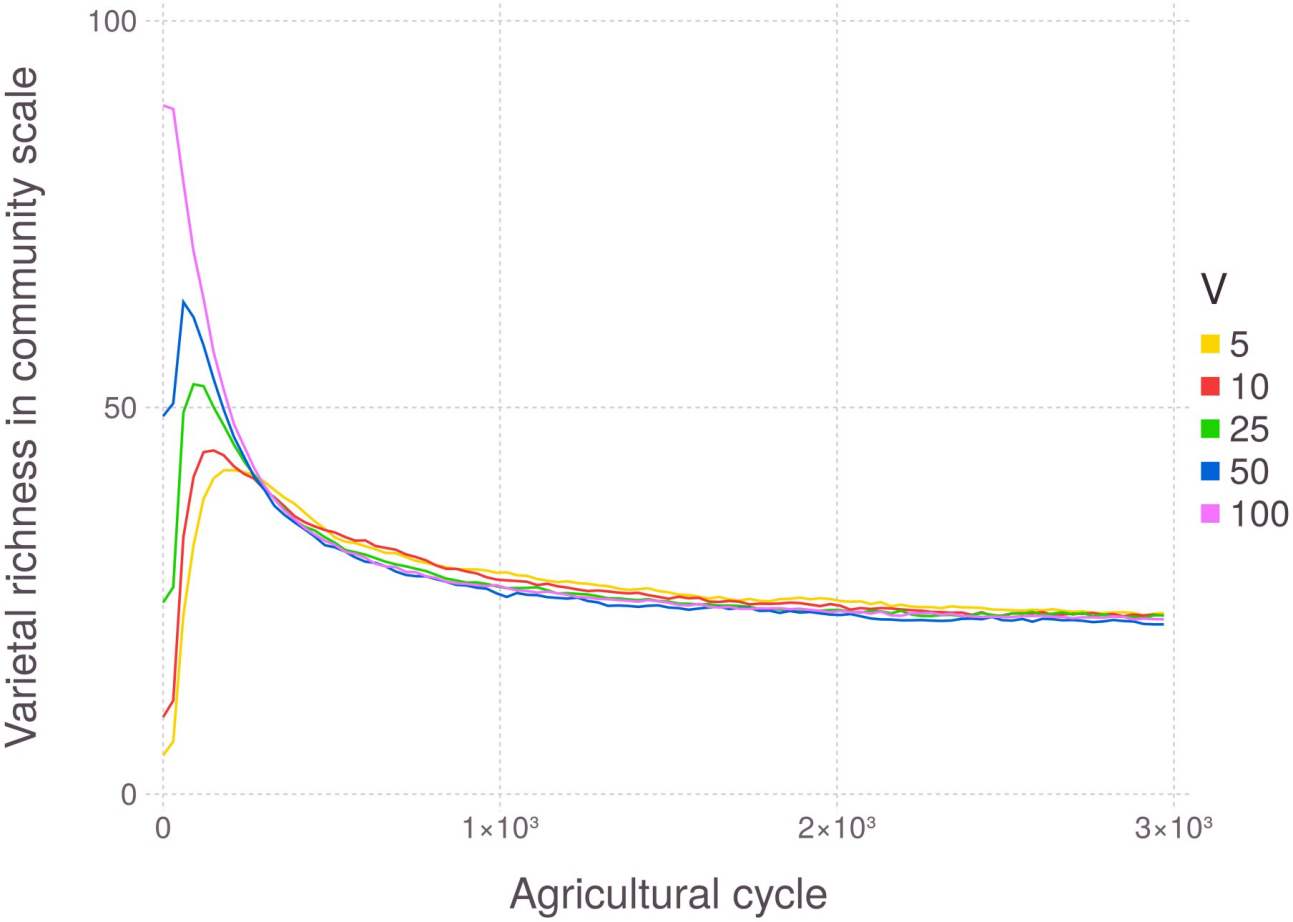
Evolution of varietal richness at the community level, with varying initial number of varieties (V). Each line represents the aggregation of 200 runs.

The model has robust behavior regarding initial condition, with varietal richness at the end of the simulation presenting independence from the initial number of varieties in the community (Fig 4). Therefore, the system and the region of stability of the model are determined by the model parameters. Although model stability was not analytically calculated, running the model for more agricultural cycles did not alter any of the diversity indexes.

**Fig 5:**
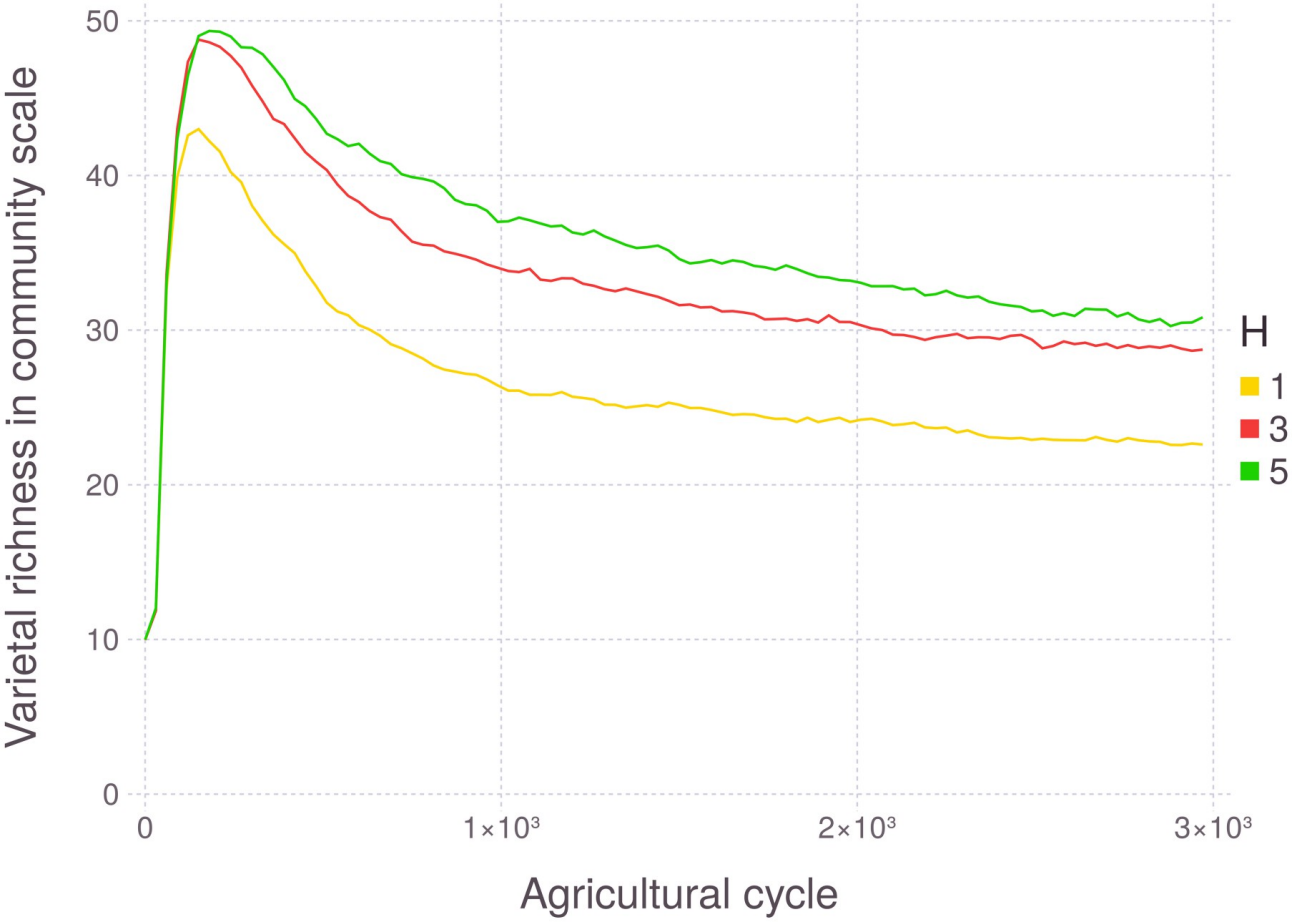
Evolution of varietal richness at the community level, with varying resource heterogeneity (H). *H* = 1 means there is only one set of resources in all plot fractions. Each line represents the aggregation of 200 runs.

As expected, community varietal richness is higher when the environment has a more heterogeneous resource distribution among plot fractions (Fig 5). In other words, varietal diversity decreases under more homogeneous environmental conditions. Resource heterogeneity implementation was analogous to [61], with contiguous rectangular regions containing each set of resources.

**Fig 6:**
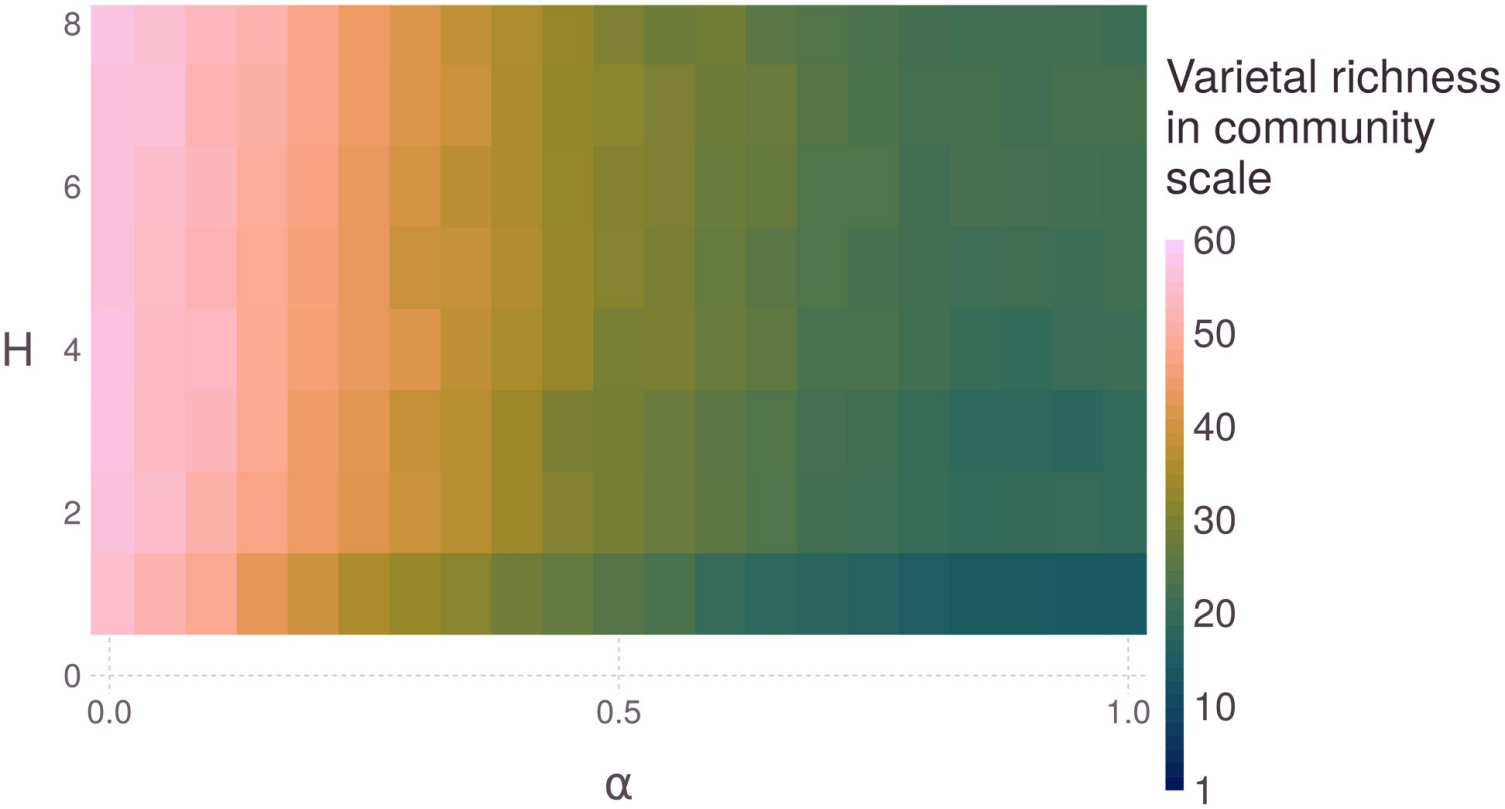
Phase diagram of the model’s final varietal richness at the community level as a function of parameters H and *α*. Parameter H is the resource heterogeneity, with *H* = 1 meaning there is only one set of resources in all plot fractions. Parameter *α* is the importance of productivity for calculating the variety score: an *α* = 0 score only considers quality fitting, and an *α* = 1 score only considers productivity. Each rectangle represents the aggregation of 100 runs for 10,000 agricultural cycles.

Equally, the parameter *α* is crucial for model dynamics and for determining the model’s region of stability (Fig 6). This parameter indicates the importance of productivity for calculating the variety score. When *α* is lower, the household will give more importance to quality fit during selection; when *α* is higher, productivity will have more importance. Letting productivity be more important—especially in more homogeneous environments—leads to few varieties being more fitted and selected by the household, therefore decreasing final varietal richness. Furthermore, resource heterogeneity has slightly greater importance when *α* is higher (Fig 6), which is also an expected result.

**Fig 7:**
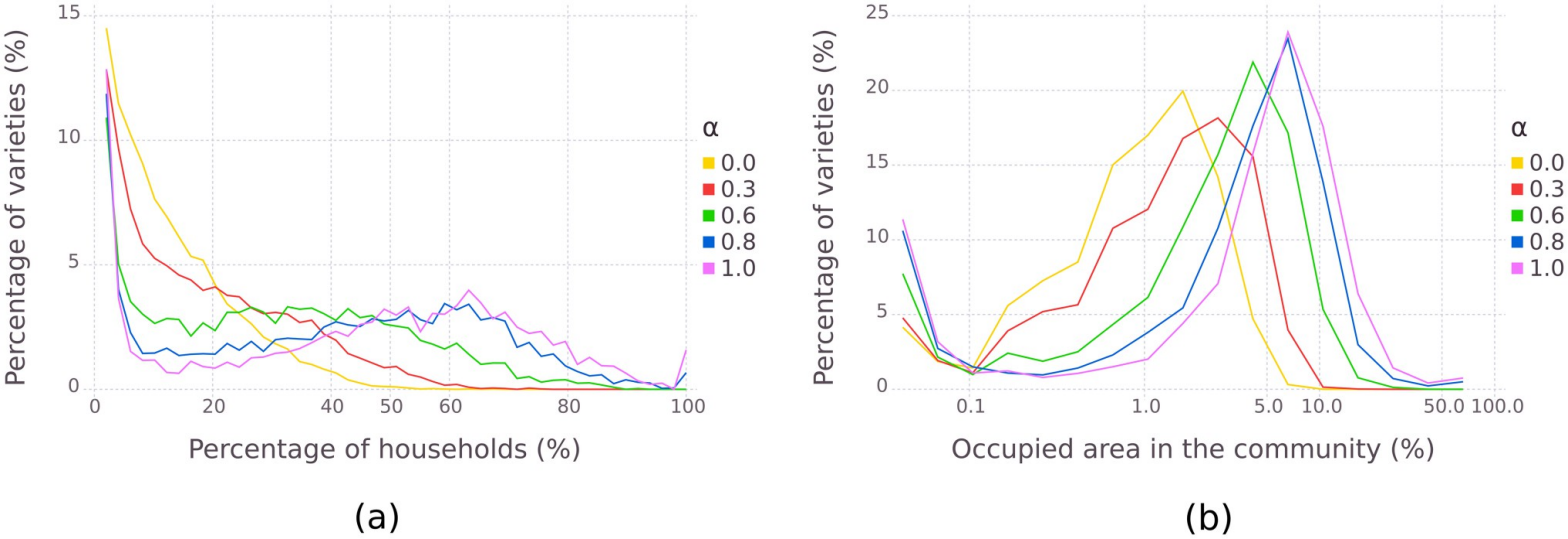
Distribution of variety evenness at the end of the simulation, with varying importance of productivity in the score (*α*). An *α* = 0 score only considers quality fitting, and an *α* = 1 score only considers productivity. Each line represents the aggregation of 200 runs. (a) Distribution of the presence of varieties in HDs. 100% indicates that the variety is in every HD of the community. (b) Distribution of the presence of varieties in community’s plot fractions.

The model shows that a system with higher *α* will select specific varieties that will be cultivated by the majority of the households (Fig 7a) and have a higher density of cultivation in the community (Fig 7b). On the other hand, focusing on quality fitting enables the system to have greater diversity, as different households have different preferences.

**Fig 8:**
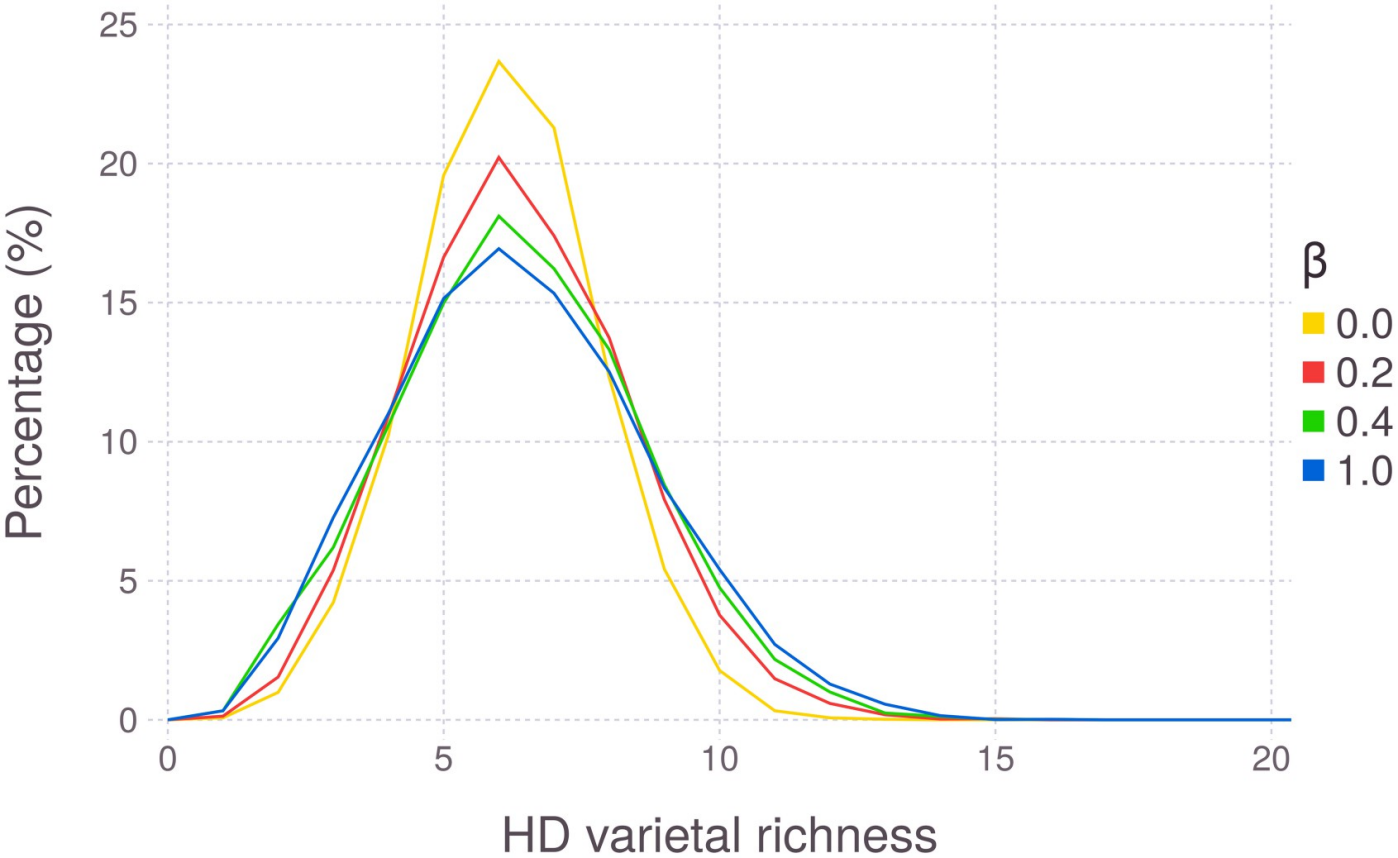
Distribution of HD varietal richness at the end of the simulation, varying the Watts-Strogatz network rewiring probability (*β*). *β* = 0 results in a regular network, and *β* = 1 results in a random network. Each line represents the aggregation of 200 runs.

A change in the household network structure affects varietal diversity. However, different from the previous parameters, the main consequence occurs at the household level and not at the community level. Network structure (or topology) encompasses how links are distributed between actors given a total number of actors and links [62]. Here it was altered by varying the rewiring probability of the Watts-Strogatz model (*β*). When *β* is higher, the network will have higher randomness. With a pure regular network (*β* = 0), the system’s final HD varietal richness distribution variance is smaller (Fig 8). Also, the presence of highly diverse HDs is more common for less structured networks (Fig 8). Varietal richness did not differ at the community level, and the average HD varietal richness went from 6.41 (for *β* = 0) to 6.14 (for *β* = 1).

The coupling of productivity and quality fit selection criteria with adequate parameters can generate behavior similar to real world data. The parameterized model produces similar community and average household varietal richness in relation to literature review data for manioc and maize (Table 2). Here, *β* diversity is the ratio between community and household richness, and the Simpson, Shannon, and Berger-Parker indexes measure diversity evenness and dominance across the community and household levels. Although the amount of data is smaller for evenness and dominance indexes, they indicate that model diversity is distributed equivalently to real world data for both manioc and maize.

**Table 2:** Comparison of diversity indexes between model values and literature review values for manioc and maize. The number in parenthesis is the standard deviation; N is the sample size.

	Model Manioc (N = 200)	Review Manioc	Model Maize (N = 200)	Review Maize
Community richness	22.7 (57)	22 (21) (N = 91)	11.5 (41)	12.0 (65) (N = 36)
Average HD richness	6.4 (9)	6.4 (63) (N = 80)	2.36 (41)	2.04 (75) (N = 65)
*β* diversity	3.57 (5)	3.03 (17) (N = 77)	4.80 (12)	4.65 (34) (N = 29)
Community Berger-Parker	0.108 (30)	0.30 (2) (N = 2)	0.20 (7)	—
Community Simpson	0.925 (27)	0.80 (12) (N = 20)	0.82 (9)	0.53 (14) (N = 3)
Community Shannon	0.89 (32)	0.75 (11) (N = 3)	0.83 (8)	—
Average HD Berger-Parker	0.375 (27)	—	0.550 (40)	—
Average HD Simpson	0.232 (24)	0.44 (17) (N = 14)	0.369 (37)	0.29 (12) (N = 4)
Average HD Shannon	0.795 (21)	0.81 (36) (N = 12)	0.839 (41)	0.74 (N = 1)

## Discussion

Despite the ecological and cultural differences among traditional agroecosystems, high varietal diversity is a common feature in many of them, suggesting an adaptive importance to maintaining diversity. Here we proposed a generative model to understand which mechanisms could generate such regularity. The results presented in the previous section show that the model is able to obtain high varietal diversity as a macro level emergent phenomenon of the system. Below we present how plot fraction, household, and community levels contribute to increase the system’s varietal diversity.

In a condition of direct optimization of agricultural productivity with uniform resource distribution, our model behaves like expected. Few varieties are more adequate, and they dominate the system. Diversity enhances in the model already at the lowest level—plot fraction –, where the biophysical component of the model is defined. The productivity of a variety depends on resource availability in the plot fraction. As expected according to niche theory [63], greater resource heterogeneity (H) increases the number and types of niches available in which different varieties can thrive, thereby corresponding to increased household and community varietal diversity. For instance, [24] showed that farmers plant different sets of manioc varieties in different soil types.

The intermediate level—household—is the most important element of the model. Most of the model dynamics are determined at this level, as all decisions of which variety to plant are made by the household. One mechanism favoring diversity at this level is the conceived selection criteria. Community diversity increases by letting less adequate varieties be present at low density in the HDs. Usually, each HD maintains a different set of less adequate varieties, which makes them more fragile while contributing to increase community richness. Empirical evidence shows the high contribution of the so-called *rare varieties* in maintaining diversity [18, 22, 23]. According to [23], Makushi farmers explain that they protect rare varieties because ‘bad’ varieties can become ‘good’ under different conditions.

Another parameter of the model, the importance of productivity in the variety score (*α*), can also considerably increase community diversity. The model uses only two factors to rank varieties: productivity and quality fit. The quality fit is a simple fit between the variety quality and the HD’s desired quality. Yet, by enabling heterogeneity in the household’s desired quality, different varieties are selected for each HD, therefore increasing both *β* diversity and community richness. Quality can be defined by cultural factors such as ethnic identity and heritage attached to a certain collection of varieties; religious or magical uses [31, 64]; or culinary traditions [25].

The three mechanisms presented above are the main promoters of varietal diversity in the model. The selection criteria show how farmer decision-making (i.e., how farmers decide which varieties to plant) that deviates from the simple rational agent optimizing productivity can enhance diversity. Also, it represents a way of including farmer’s intentionality to have more varieties into dynamics modeling. Resource heterogeneity and quality fit show how spatial (biological) and cultural (heterogeneity) agents can increase varietal richness. The heterogeneity implemented at the micro level has an important role in macro level behavior. This is a benefit of agent-based modeling that can help to explain new phenomena that cannot be explained by homogeneous average agents [32, 33].

Within this model, the social network acts as an integrative mechanism at the community level. It determines how households interact with each other and are responsible for socializing the locally created varieties. There is still an open question of how different seed exchange network structures would influence varietal diversity and what structures are better to promote diversity [46, 57, 65, 66]. Although this work did not focus on this issue, we illustrate that network structure is important to determine how varietal diversity is distributed along the household and community levels. However, different from what was expected, this distribution did not have a great influence on community richness. We present two explanations for that: firstly, the network structure had no effect upon final community richness; however, it impacted transient community richness, with more random networks taking longer to stabilize, while more structured networks stabilize faster. Therefore, the network structure effect seems to have a greater impact on the short term. Secondly, the effect of network structure is usually more prominent in larger networks [62], with a likely greater impact on the system’s varietal diversity. More research is needed to answer this important open question.

Here we presented a generalist model of an agricultural socioecological system with the aim of obtaining high varietal diversity as an emergent phenomenon caused by heterogeneous locally interacting actors. The model was able to produce varietal richness at both the community and household levels that are similar to that of *Manihot esculenta* and *Zea mays* case studies. We also explored how plot fraction, household, and community levels can shape agricultural diversity, altering varietal richness and evenness.

However, models are always a simplification of the real world and, as such, have limitations. We did not include some factors that are certainly important for farmer’s decision-making (risk aversion, local availability of resources, policy interventions, and market economy) and for productivity assessment (seasonality, biotic stress). Nevertheless, by considering a few factors, we were still able to design a model which qualitatively reproduces empirical data. To answer how well we accomplished our goal, we compared simulated and empirical data, assuring some basic internal consistency constraints were followed. We hope such a model can help to explain, in terms of a few factors, some features of such complex socioecological systems.

Traditional agricultural systems are key to global food security in face of climate change. By modeling the underlying dynamics that influence the production of agrobiodiversity, the model can contribute to the development of future social-ecological models and indicators of agrobiodiversity, as well as policies and incentives to protect complex traditional agricultural socioecological systems.

## Supporting information

S1 Text Detailed model description. Detailed description of model’s dynamics and parameters.
(PDF)

S1 Dataset Case studies dataset. Manioc (*Manihot esculenta*) and maize (*Zea mays*) varietal diversity dataset.
(XLSX)
