# Supplementary material for: An integrated model to study varietal diversity in traditional agroecosystems
Source: PLoS One. 2022 Jan 28;17(1):e0263064. doi: 10.1371/journal.pone.0263064 (PMC8797245; doi:10.1371/journal.pone.0263064)
Supplement: S1 Text — Detailed description of model’s dynamics and parameters. (PDF) [file pone.0263064.s001.pdf]

Supplementary Material I

This supplementary material shows a detailed description of the model presented in the article *An integrated model to study varietal diversity in traditional agroecosystems*. The model was developed in C++ and the source code is available at https://github.com/vitorhirata/project-agrobio.

1. Models dynamics

Here we describe the model initialization and five steps that occur in one model's cycle. During the description of the dynamics, some model parameters are referenced and can be consulted in the last section.

1.1. Initial Condition

The model begins by sorting varieties among the plot fractions. It initiates with V varieties available in the community and for each household V_{HD} varieties are randomly selected to be available for the respective household. Then, for each household's plot fraction one variety of the ones available to the household is selected. Each variety is created with quality and half-saturation constants following a normal distribution centered in 0.5 with 0.15 standard variation. A variety with the same quality - within the range of 0.05 for manioc and 0.1 for maize - and the same three half-saturation constants - within the range of 0.1 for manioc and 0.2 for maize - is considered to be the same variety. The household's desired quality is also chosen randomly, based on a normal distribution with a mean of 0.5 and a standard deviation of 0.1.

Each plot fraction has a certain amount of three biochemical resources. These values are initialized based on a normal distribution with a mean of 0.6 and a standard deviation of 0.2. Depending on the *ambient heterogeneity* (H), each plot fraction will have different sets of resources or have the same one in all plot fractions. The amount of resources in each plot fraction is constant during the simulation.

The previous household network is defined in the model initialization and remains constant through the simulation. It is calculated following the Watts-Strogatz algorithm, with an initial degree of the regular network of 4 and a rewiring probability (β) of 0.2.

1.2. Individuals survivability

Individual varieties can die and this is the main way a variety can get extinct in the model. It represents both the natural death - lack of soil nutrients, water, or extreme temperature conditions - and human origin - lack of ability to handle the cultivar, problems in planning seed quantity, losing seeds. In the model this process happens stochastically, therefore, for each plot fraction there is a death probability equal to:

$$d_{v,pf} = d_b - d_c P_{v,pf},$$

where $d_{v,pf}$ is the death probability of the variety *v* in the plot fraction *pf* and $P_{v,pf}$ is the productivity - given by the Monod Equation - of the variety *v* in the plot fraction *pf*; d_b is the base death probability and d_c is the death productivity linear coefficient, both are parameters of the model, see Table S1.2 for their values.

1.3. Internal Selection

The internal selection comprehends the complex process of farming decision making, therefore is the most complicated step. In this step, the household changes up to twenty plot fraction varieties. To decide which varieties production will be increased or decreased the household uses the varieties score. The score considers a biological factor, given by the Monod Equation, and a cultural factor, given by the quality fit. These factors are then coupled with a parameter α. The score of a variety *v* in a household *HD* ($s_{v,HD}$) is defined as:

$$s_{v,HD} = \alpha P_{v,HD} + (1 - \alpha)Q_{v,HD},$$

where $P_{v,HD}$ is the average productivity of variety *v* in the household *HD*, and $Q_{v,HD}$ is the quality fit between variety *v* quality and household *HD* desired quality.

In the internal selection step, each household tries to minimize the difference between the desired variety density ($\mu^d_{v,HD}$) and the real variety density ($\mu^r_{v,HD}$) for all varieties, i.e. to minimize $\Delta\mu_{v,HD} = \mu^d_{v,HD} - \mu^r_{v,HD}$. The result measures the household HD desire to increase (or decrease) the density of the variety *v*. The household can change its past production from 1 to 20 times - this is chosen randomly based on a uniform distribution - and each change in production will involve removing an individual variety that has a lower $\Delta\mu_{v,HD}$ and adding one individual of a variety that has the higher $\Delta\mu_{v,HD}$.

The desired density of the variety depends on the difference between the variety's score and the average score of the varieties existent in the household. It is also normalized so that it stays between the range [0,1]. The values of $s_{v,HD} - \overline{s_{HD}}$ are very low and usually are in the interval [-0.2, 0.2]. Due to this fact, a sigmoidal function (Fig. SI.1.) is added to increase the argument around this interval. Therefore, the desired density is written as:

$$\mu^d_{v,HD} = \frac{f(s_{v,HD} - \overline{s_{HD}})}{\sum_v f(s_{v,HD} - \overline{s_{HD}})},$$

where $f(\Delta P) = 0.5 + \frac{S\Delta P}{\sqrt{1+(2S\Delta P)^2}}$ is a sigmoidal function used to renormalize ΔP, and S is a parameter of the model that determines the slop of f (see Table S1.2 to see its value).

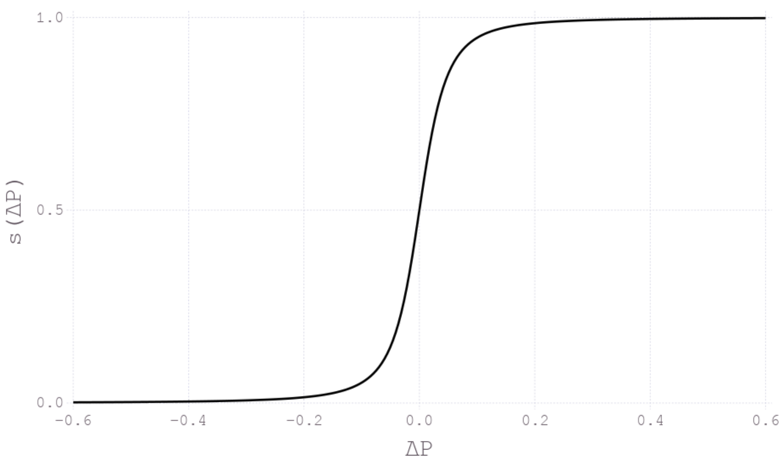

Figure SI.1: Plot of the renormalization function used for internal selection, using parameters for the manioc. Where ΔP = $p_{v,HD} - \overline{p_{HD}}$.

The real variety density is simply the number of plot fractions occupied by the variety in the household ($n_{v,HD}$) divided by the household total number of plot fractions (N_{HD}), a constant number. Therefore, the final equation for $\Delta\mu_{v,HD}$ is:

$$\Delta\mu_{v,HD} = \mu^{d}_{v,HD} - \mu^{r}_{v,HD} = \frac{f(s_{v,HD} - \overline{s_{HD}})}{\sum_{v} f(s_{v,HD} - \overline{s_{HD}})} - \frac{n_{v,HD}}{N_{HD}},$$

1.4. Substitution of dead individuals

In this step, the variety with higher $\Delta\mu_{v,HD}$ in the household is placed in all the plot fractions that are without a variety due to the model's first step.

1.5. Exchange between households

All exchanges happen within the previously defined network, representing the kinship, godparenting and neighborhood networks. In the model, each household has a base exchange probability and this is multiplied by the household score, which is the average of owned varieties score weighted by the density of each variety. When an exchange happens the household takes a variety selected stochastically, with the probability of selection being the real variety density ($\mu^{r}_{v,HD}$) of the selected household, and then places it on one of its plot fractions, which is chosen randomly.

1.6. Emergence of new varieties

New varieties can arise in two ways, by sexual reproduction between existing varieties or by exchanges that happen outside the community. Varieties emerging from sexual reproduction have the average value of the characteristics - half-saturation constants and quality - of two previously existing varieties, with the addition of noise with maximum module corresponding to the traits mating deviation, a parameter of the model. Varieties

emerging from outside the community are considered completely new, so new characteristics are sorted as described in the initial condition.

2. Parameters

We have two versions of the model's parameters, one for fitting the bibliographic review of manioc and another one for maize. The parameters were chosen keeping reasonable boundaries taken from the literature and fitting the case studies for each species, with no empirical parameter used. In Table SI.1 some base parameters are presented; they set constraints to the model and are not very influential on model behavior. Table SI.2 presents more important parameters that can drive the model stability area and behavior, with two different versions for each species.

Table S1.1: Base model parameters description and values.

Name	Description	Value
Community size	Number of plot fractions in the community	2401 (49x49)
Household size (N_{HD})	Number of plot fractions owned by a household.	49 (7x7)
Number of households	Number of households in the community	49
Number of agricultural cycles	Number of agricultural cycles in a run of the model	3000
Initial number of varieties in the community (V)	Maximum number of initial varieties in the community	10
Initial number of varieties per household (V_{HD})	Maximum number of initial varieties in the household	5
Number of resources	Number of different biochemical resources	3

Table S1.2: Main model's parameters, with respective descriptions and values.

Name	Description	Value Manioc	Value Maize
Importance of productivity (α)	Importance given to productivity in the calculation of variety score	0.6	0.6
Ambient heterogeneity	Number of different sets of resources.	1	1

(H)	Alternatively, number of different habitats in the community		
Base exchange probability	Base probability that two households are going to perform an exchange (per pair of households)	0.32	0.2
Watts-Strogatz rewiring probability (β)	Rewiring probability of two given nodes, according to the Watts-Strogatz algorithm	0.2	0.2
Probability of new random variety	Probability that a new random variety emerges (per household)	0.006	0.006
Probability of new mating variety	Probability that a new variety emerges from the crossing of two previously existing varieties (per household)	0.054	0.24
Traits matting deviation	Maximum deviation that can happen in the variety traits in each crossing	0.005	0.005
Slop of sigmoid function	Slop of sigmoid function (f) used to renormalize ΔP	10	20
Death probability	Probability that one individual variety will not survive (per plot fraction) given its productivity in the plot fraction is $P_{v,pf}$	0.2 - 0.17 * $P_{v,pf}$	0.35 - 0.17 * $P_{v,pf}$
